# Knowledge and Practice towards Alcohol Consumption in a Sample of University Students

**DOI:** 10.3390/ijerph18189528

**Published:** 2021-09-10

**Authors:** Marisa Patrizia Messina, Gemma Battagliese, Alessio D’Angelo, Rosaria Ciccarelli, Fabiola Pisciotta, Luigi Tramonte, Marco Fiore, Giampiero Ferraguti, Mario Vitali, Mauro Ceccanti

**Affiliations:** 1 Department of Gynecology, Obstetrics and Urology, Sapienza University of Rome, 00185 Rome, Italy; marisapatrizia.messina@uniroma1.it (M.P.M.); alessio.dangelo96@gmail.com (A.D.); 2 Centro di Riferimento Alcologico della Regione Lazio, Mental Health Department, ASL Roma 1, 00185 Rome, Italy; gemma.battagliese@aslroma1.it; 3 Società Italiana per il Trattamento dell’Alcolismo e le sue Complicanze (SITAC), ASL Roma1, Sapienza University of Rome, 00185 Rome, Italy; r.ciccarelli@evodevo.it (R.C.); fabiola.pisciotta@uniroma1.it (F.P.); 4 Faculty of Medicine and Dentistry, Sapienza University of Rome, 00185 Rome, Italy; tramonte.1634054@studenti.uniroma1.it; 5 Institute of Biochemistry and Cell Biology (IBCN-CNR), 00185 Rome, Italy; marco.fiore@cnr.it; 6 Department of Experimental Medicine, Sapienza University of Rome, 00185 Rome, Italy; giampiero.ferraguti@uniroma1.it; 7 ASUR Marche, AV4, 60122 Ancona, Italy; vitalimario@yahoo.it

**Keywords:** FASD, abuse, alcohol, student, university, medical faculty

## Abstract

**Objective:** Alcohol affects many human systems and is involved in the pathogenesis of other diseases. Particular attention must be paid to alcohol consumption among young people. It has been shown that 25% of young people’s deaths are attributable to alcohol, and around 35 million people aged over 11 had consumed at least one alcoholic beverage in 2015. **Study Design:** Young people aged 18–24 were the most vulnerable to binge drinking in Italy, and 50.6% of teenagers drunk alcohol. Only a few studies in the literature have investigated those habits in university students. This study aims to examine alcohol use habits in a population of university students in Italy. **Methods:** Between 2018 and 2019, an anonymous online questionnaire was randomly sent to university students from 17 different universities in a network of research centres to study alcohol use disorders. The survey included socio-demographic information, questions about alcohol use, knowledge about alcohol consumption, and related risks. Used questionnaires were the Alcohol Use Disorders Identification Test-Consumption (AUDIT-C) and the Drinking Motive Questionnaire-Revised (DMQ-R). **Results:** the AUDIT-C revealed that 53.3% of students were high-risk drinkers. Regarding binge drinking habits, 13.1% of students admitted to binge drinking behavior at least once a month. In our sample, male students are more likely to be low-risk drinkers than female peers (*p* < 0.008). Students from northern Italy are more likely to be high-risk drinkers (*p* = 0.003). Beer (65.9%) and wine (60.9%) were the most consumed alcoholic beverages. The most common places to drink alcohol were pubs (85.5%). The most likely motivations to drink alcohol were enhancement (40.43%), social (38.39%), coping (15.63%), and social pressure or conformity (5.55%). Only 43.8% of participants reported having attended an educational course on alcohol. **Conclusions:** University students were not fully aware of the implications of alcohol misuse and will be part of the adult society as critical figures and future leaders. It is imperative to inform students about alcohol consumption risks and investigate the motivations to drink. Stress, anxiety, and social pressure are only a few issues young people are exposed to. Special attention must be paid to young people and their coping strategies that involve substance abuse by using educative, preventive, and motivational approaches.

## 1. Introduction

Although a large body of evidence about the detrimental effects of alcohol on behavioral [1,2,3,4,5,6] and physical health [7,8,9,10,11,12] has been provided, a significant amount of people in Europe are still drinking [13] and it is still considered part of a Mediterranean diet [14,15,16,17,18,19,20]. The most affected human systems are the nervous, digestive, and cardiovascular systems [12,21,22]. The International Agency for Research on Cancer (IARC) has determined that alcohol consumption is causally related to the oral cavity, oropharyngeal, hypopharyngeal, esophageal, colon, rectal, laryngeal, liver and intrahepatic bile duct, and breast cancers [23,24,25,26,27,28,29,30,31]. Chronic alcohol consumption has been observed to lead to insulin resistance, resulting in a higher risk of diabetes mellitus by disrupting glucose homeostasis in drinkers [32,33]. Alcohol has a clear impact on hemorrhagic strokes (causing 9.5% of all hemorrhagic stroke deaths), hypertensive heart diseases (7.4% of all hypertension deaths), cardiomyopathies (6.8% of all cardiomyopathy deaths), ischemic heart diseases (2.7% of all ischemic heart disease deaths), and other cardiovascular disorders [12,34,35,36,37,38,39,40]. Moreover, alcohol can affect the innate and the acquired immune systems and may increase vulnerability to infectious diseases [21,22,41,42]. Alcohol consumption was proved to push people into adventurous sexual behaviors and increase the likelihood of unsafe sex, contributing to sexually transmitted diseases [42,43]. There is also evidence that, in the alcohol-use-disorder population, 50.3% of patients had psychiatric comorbidity during their lifetime [44,45,46,47,48,49,50].

Another crucial point is that alcohol consumption during pregnancy has been shown to provoke in newborns fetal alcohol spectrum disorders (FASD), the leading cause of mental retardation in western countries, and in the most severe case (FAS, fetal alcohol syndrome), physical dysmorphology [51,52,53,54] as shown in humans and FASD animal models [55,56,57,58,59,60,61,62]. The social costs of such preventable conditions are still countless. The lifetime cost per child with FASD in the US was $2,000,000 in 2002 [63].

Particular attention must be paid to alcohol consumption among young people [4,49,64]. According to the WHO (2014), 25% of young people’s deaths are attributable to alcohol. The Italian Ministry of Health reported that in 2015 [65], around 35 million people aged over 11 had consumed at least one alcoholic beverage, with a great preponderance of males over females (77.9% vs. 52.0%). In particular, the age group between 11 and 24 years old shows a widespread custom of drinking between meals, with a frequency of at least once a week, and often binge drinking. In 2015, around 15.6% of young people aged 18 and 24 experienced binge drinking (22.2% males, 8.6% females). This percentage reached the 17% in 2016 (21.8% males and 11.7% females) [66]. Young people aged 18 and 24 are the most vulnerable to binge drinking in Italy [67]. 

Although it is legally forbidden to sell alcoholic beverages to minors in Italy (law n.189 of 2012), the Adolescent Observatory of Blue Telephone and DoxaKids [68] confirmed that 50.6% of respondent teenagers, aged 11 to 19 years old, happened to drink alcohol, and 49.9% got drunk at least once.

These frightening data indicate that alcohol abuse is widespread among young people exposed to both short- and long-lasting repercussions of such lifestyle over health. 

However, only a few studies in the literature investigated those habits in young people, such as university students, and this is the first study on the awareness of alcohol-related risks in students from different universities in Italy. After investigating alcohol consumption, knowledge, and practices among adult healthcare professionals [54,69], our study group examined alcohol use habits in a sample of university students in Italy by using an anonymous online questionnaire.

## 2. Methods

### 2.1. Subjects’ Recruitment

Between 2018 and 2019, an anonymous online questionnaire was randomly sent via web to university students from 17 different universities belonging to a network of research centres aimed at studying alcohol use disorders and coordinated by SITAC (Società Italiana per il Trattamento dell’Alcolismo e le sue Complicanze). These university students of different nationalities were geographically distributed between the northern, central, and southern areas. We randomly sent 2,835 questionnaires via the web to a sample of Italian university students and 1,928 returned (68.01%) of these were returned. This survey included socio-demographic information, questions about alcohol use and knowledge towards alcohol consumption, and related risks. The used questionnaire was the Alcohol Use Disorders Identification Test-Consumption (AUDIT-C). Motivations for drinking were investigated through the Drinking Motive Questionnaire-Revised (DMQ-R). The system used did not permit cheating attempts, for example, consulting online resources to answer the questions. Moreover, students were also asked if they ever attended a lesson or any other training tutorial on topics related to the risks of alcohol consumption. The ASL Roma 1 ethical committee approved the study (Prot. N. 337/CE Lazio 1), each participant signed informed consent, and all the study procedures followed the Helsinki Declaration of 1975, as revised in 1983, for human experimentation.

### 2.2. AUDIT-C

The AUDIT-C (Alcohol Use Disorders Identification Test-Consumption) is a validated tool [70] to assess alcohol consumption through three short questions that estimate alcohol consumption in a standard, meaningful, and non-judgmental manner. The total score from these questions indicates the health risks and addresses the conversations about alcohol habits. The AUDIT-C is a shortened version [71] of the 10-item AUDIT tool developed in a WHO collaborative project [72] and has been extensively used and researched. 

The score for each question is from 0 to 4 on a five-point scale. The score of the single items summed makes the total score maximum of 12. The optimal AUDIT-C thresholds for alcohol misuse in the USA are ≥4 points for men and ≥3 for women [73,74].

The AUDIT questions/scores and a chart illustrating the approximate number of standard drinks in different alcoholic beverages are available online https://www.drugabuse.gov/sites/default/files/files/AUDIT.pdf (accessed on 8 August 2021) Information about the sensitivity and specificity of the test can be found at the following address: https://pubs.niaaa.nih.gov/publications/arh25-3/204-209.htm (accessed on 8 August 2021).

### 2.3. Drinking Motives Questionnaire (DMQ-R)

The Drinking Motives Questionnaire-Revised (DMQ-R) [75] is a self-administered questionnaire with 12 items. The test is reflecting the frequency of occurrence of specific drinking motives that evaluates four possible classes of drinking motives: internal positive reinforcement (Enhancement), external positive reinforcement (Social), internal negative reinforcement (Coping), and external negative reinforcement (Conformity). DMQ-R is fully displayed in Table 1.

### 2.4. Knowledge towards Alcohol Consumption

A 14-items questionnaire was used to investigate the knowledge of those professionals towards alcohol consumption (Table 2). The 14-items questionnaire was developed by SITAC (Società Italiana per il Trattamento dell’Alcolismo e le sue Complicanze-Italian Society for the Treatment of Alcoholism and its Complications) and is used in the network of Italian research centres coordinated by SITAC for the study of alcohol use disorders.

### 2.5. Statistical Analysis

ANOVA with Tamhane correction was used to analyse age and gender factors according to methods previously described [76,77,78]. Post-hoc comparisons were analysed by the Tukey’s HSD test. The Chi squared test for linear trend was used to analyse the answers to the fourteen questions concerning the “Knowledge Towards Alcohol Consumption”. The SPSS software was used to perform all statistical analyses (version 21; IBM SPSS Statistics, Chicago, IL, USA).

## 3. Results

### 3.1. General Description of the Recruited Subjects

The characteristics of subjects included in the study are shown in Table 3. All the participants were Italian, but the 1% were Italian speaking strangers. The sample’s mean age was 21.8 ± 2.4 years, ranging from 18 to 26 years old. The sample was composed mainly of male participants (79.9% vs 20.1%). The higher number of students were located in central Italy (42%).

### 3.2. Drinking Habits

The AUDIT-C assessed drinking habits, and it revealed only 7.0% of no drinkers, 39.5% of low-risk drinkers, and 53.3% of high-risk drinkers (Table 4). Concerning binge drinking habits, 37.2% reported having never assumed more than five drinks on a single occasion. The 0.6% of students admitted binge drinking behavior at least once a day, 3.3% at least once a week, 13.1% once a month, 45.5% less than once a month. Significant differences were found between women and men in alcohol consumption: male students were more likely to be low-risk drinkers than female peers (*p* < 0.008). Moreover, students from northern Italy are more likely to be high-risk drinkers (*p* = 0.003).

It was also asked what kind of alcoholic beverages they used to drink and in what locations: beer and wine were the most consumed alcoholic beverages (65.9% and 60.9%, respectively), followed by cocktails (58.2%), spirits (31.0%), and other alcoholic drinks (18.4%). The most common places to drink alcohol were pubs (85.5%) and parties (69.2%), followed by friends’ houses (53.7%), the home (34.1%), disco (32.1%), and others (11%) (Table 3). 

The most likely motivations to drink alcohol were investigated through the DMQ-R questions: 38.39 % used to drink to be sociable or to celebrate parties (social), 15.63 % used to drink to forget about problems (coping), the 40.43 used to drink to feel better or to be able to do things otherwise impossible (enhancement), the 5.55 % used to drink because other people do (social pressure or conformity) (Table 3). ANOVA demonstrated that high-risk drinkers drank a higher amount of alcohol compared to the low-risk drinker for coping (F = 79.9; *p* < 0.001)*,* social (F = 95.6; *p* < 0.001), and enhancement (F = 81.9; *p* < 0.001). On the other hand, lower-risk drinkers drank more for conformity (F = 7.4; *p* < 0.001) than high-risk drinkers.

### 3.3. Knowledge towards Alcohol Consumption

Only 43.8% of participants reported having attended a course about alcohol. The SITAC 14-item questionnaire was used to investigate the knowledge of those professionals towards alcohol consumption (Table 2). Table 5 shows the results about knowledge towards alcohol. 

The most incorrect answers were given to items 7 and 10, “*Where is the alcohol located on the scale of cancerogenic substances?*” and “*In Alcohology, what is meant by the age of risk consumption?*” scoring low values for correct answers. On the other hand, the correct answers were given to item 1, “*Is in Italy requested a minimum age for the administration and sale of alcohol?*” and item 6, “*What is Fetal Alcohol Syndrome?*”. Quite interestingly, the at-risk group of drinkers displayed better performances on items 5 “*Which is the alcoholic level in the blood (grams) beyond which a novel driver or a person under 21 years old could be sanctioned?*”, 8 “*Which pathology can chronic alcohol abuse cause?*”, 11 “*How many alcoholic units have to be consumed to define ’Heavy Drinking’?*” and 12 “*In Alcohology, what is meant by year of risk consumption?*” but a low percentage of correct answers on item 7 “*Where is the alcohol located on the scale of cancerogenic substances?*”. To confirm the displayed knowledge of the at-risk group in some answers, the Chi-squared test for linear trend for all answers does not differ significantly from linearity but for the answers to the questions number 5 and 12 significant values emerged for the at-risk group, Pearson’s χ^2^ = 9.82, dF(2), *p* = 0.0072; χ^2^ = 11.51, dF(2) *p* = 0.0032.

## 4. Discussions

Investigating alcohol consumption is critical for the scientific community and health institutions [12,20]. Its repercussions on health have been extensively provided, and its social and economic implications are still downrated [79]. Very few studies were found about alcohol consumption among university students: a systematic review [80] examined 29 scientific articles investigating alcohol consumption by Irish and English university students. This study showed that most students are high-risk drinkers, and about 20% of students declare high alcohol consumption during weekdays. Our data substantially agrees with the English data, confirming that most students in Italy are high-risk drinkers (53.3%). 

Davoren and colleagues [81] identified four categories of drinkers among university students: (1) The controlled drinkers, (2) the hedonists; (3) students who consume alcohol in social situations because influenced by their peers; (4) the uncontrolled drinkers to handle adverse conditions. 

To the best of our knowledge, this is the first web interview-based study on the knowledge about alcohol-related risks (including FASD) on students from different universities of Italy. A research conducted in 2011 by the University of Camerino [82] reported that among 345 university students attending different faculties, only 14.4% stated abstinence. However, among the students who were assessed to be regular consumers, a percentage of 34.4% of males and 14.9% of females were estimated to consume alcohol several times a week or per day. Furthermore, 50.4% of the participants already had illegal psychotropic substances, and 53.1% were smokers. 

Another study [83] conducted in Brazil on 281 nursing students revealed that 90% of the students had consumed alcohol at least once in life and the age of first-time drinking was 15.4 years (20.6). It has also been hypothesized that the amount of stress university students are put under could enhance alcohol consumption in this population [84]. It has been reported that healthcare student’s positions and beliefs about alcohol are still unclear and probably stereotyped [85]. Furthermore, specific clinical placements in alcohol rehab facilities were proved effective in sensitizing students about alcohol abuse disorder [86].

Male students are more likely to be low-risk drinkers than females, and students from northern Italy usually drink more. Our data are substantially in agreement with international data, confirming that most students are high-risk drinkers. Although, no significant association between alcohol consumption and age was found. Our sample students drink beer and wine predominately in bars and pubs and generally not close to meals. A significant percentage of high-risk drinkers consume alcoholic drinks to manage negative emotions (coping). In contrast, low-risk drinkers are more likely to drink to feel part of the group (social), showing better ability to manage their consumption. 

Although our system could not enable cheating attempts, only 50% of the analysed sample answered the knowledge questions correctly demonstrating that students of the present cohort aren’t sufficiently informed. We do want to stress the point that our data clearly show no differences among faculties. Being part of the medical area or healthcare professions is not enough to provide efficient information about the risks of unhealthy lifestyles and acquired habits. However, we have demonstrated an association between the consumption levels detected with the Audit-C and a more correct response to questions relating to the knowledge of the years at risk and the blood alcohol levels that can be sanctioned. Therefore, the mere knowledge of the effects of alcohol is not sufficient to prevent the onset of alcohol use disorders. The results of the Drinking Motives Questionnaire (DMQ-R) show that most of the students in the sample examined also drink for a strong inner discomfort to overcome their difficulties (15.63% used to drink for coping, 40.43% used to drink to feel better, for enhancement. About 45% then drink for compliance or social pressures. These results provide indications for a better preventive action that must be addressed not only to the knowledge of alcohol but above all to intervene precociously on the sources of stress that cause the discomfort that occurs in adolescence and grows over time Finally, particular attention must be paid to the influence that the environment can exert through direct and indirect pressures.

## 5. Implications

The main implication of this study is that the inclusion of information on alcohol risk in the educational curricula of all university degree courses is worth considering, with specific reference to addiction to alcohol, psychoactive substances and addictive behaviors. It is important to update the current methods of intervention through the proper training of students with the aim of creating professional figures capable of adequately addressing issues connected with alcohol-related problems.

## 6. Conclusions

University students are going to be part of the adult society as critical figures and future leaders. It is imperative to inform students about alcohol consumption risks and investigate their motivations to drink. This is particularly important for future health professionals who could develop problems associated with alcohol abuse. Stress, anxiety, and social pressure are only a few of the young people are exposed to. Special attention must be paid to young people and their coping strategies that involve substance abuse. On the other hand, legal restrictions are not sufficient to reduce alcohol use in young people who have easy access to alcohol. Educative, preventive, and motivational approaches to reduce alcohol abuse are urgently needed

## Figures and Tables

**Table 1:** Classes of Drinking Motives and Questions.

Classes of Drinking Motives	Questions
**Enhancement**	*Because I like the feeling;* *To obtain something;* *Because I think it’s fun;* *To please me.*
**Social**	*Because it helps to enjoy a party;* *Because it makes social gatherings more fun;* *Because it improves parties.*
**Coping**	*Because it helps me when I am sad or nervous;* *Because it cheers me up when I am in a bad mood;* *To forget about problems.*
**Conformity**	*To fit with the group of friends I like;* *To please other people;* *To not feel left out.*

**Table 2:** 14 items question on alcohol and FASD.

***Item 1***	*Has Italy requested a minimum age for the administration and sale of alcohol?*
***Item 2***	*What are the daily limits of alcohol consumption, according to the World Health Organization? How many glasses, shots, depending on the drink) beyond which it becomes dangerous to health? (For men)*
***Item 3***	*What are the daily limits of alcohol consumption, according to the World Health Organization? How many glasses, shots, depending on the drink) beyond which it becomes dangerous to health? (For women)*
***Item 4***	*How are many alcoholic units (AU) present in 660ml of beer?*
***Item 5***	*Which is the alcoholic level in the blood (grams) beyond which a novel driver or a person under 21 years old could be sanctioned?*
***Item 6***	*What is Fetal Alcohol Syndrome?*
***Item 7***	*Where is the alcohol located on the scale of cancerogenic substances?*
***Item 8***	*Which pathology can chronic alcohol abuse cause?*
***Item 9***	*Where does the alcohol absorption main occur?*
***Item 10***	In *Alcohology, what is meant by the age of risk consumption?*
***Item 11***	*How many alcoholic units have to be consumed to define ’Heavy Drinking’?*
***Item 12***	In *Alcohology, what is meant by year of risk consumption?*
***Item 13***	*If the person realized where the problem is and starts seriously thinking to a resolution, he would be in the motivational stage of Action; Determination; Maintenance; Contemplation?*
***Item 14***	*Where does alcohol induce significant damage?*

**Table 3:** Characteristics of study population: Gender, Age, Location and Faculties. Humanities (Letters, Philosophy, DAMS, Tourism, Languages, Scenography, Communication, Training, Goods cultural, Political Science, Sociology, Anthropology), Medical studies (Medicine and Dentistry); Scientific and Technological Studies (Engineering, Graphics, Architecture, Chemistry for Pharmaceutical Technologies, Physics, Agriculture, Design, Geology, Mathematics, Biology, Biotechnology, Veterinary, Chemistry, Pharmacy, Statistics, Optics, Oenology, Sciences Motor, Aviation); Healthcare studies (Nursing, Physiotherapy, Psychology, Obstetrics, Dental Hygiene, Radiology, Neuropsychomotricity, and Rehabilitation); Economic studies (Economy, Marketing, management, Banking Sciences, Finance); Legal studies (Law and Legal Services); Artistic studies (Academy of Fine Arts, Multimedia Arts, and Conservatory).

*Characteristics*	*Data - % - n*
***Gender***	
*Males*	79.9% - 1541
*Females*	20.1% - 387
***Age***	21.8 ± 2.4 years
***Geographical Distribution***	
*Northern Italy*	38.8% - 766
*Central Italy*	42% - 809
*Southern Italy*	12.7% - 244
*Not disclosed*	4.7% - 90
*Foreign Country*	1% - 19
***Faculty***	
*Humanities*	25.7% - 496
*Medical studies*	25.1% - 484
*Scientific and Technological studies*	23.4% - 451
*Healthcare studies*	12.2% - 236
*Economic studies*	7.8% - 150
*Legal studies*	3.5% - 67
*Artistic studies*	0.7% - 13
*Not disclosed*	1.6% - 31

**Table 4:** Drinking Risks, Alcoholic Beverages, Drinking Locations and Drinking Motivations.

Drinking Risks	%	Alcoholic Beverages	%	Drinking Locations	%	Drinking Motivations	%
**No**							
**Drinkers**	*7*			Pub	*85.5*		
*Men*	*4.9*						
*Women*	*7.5*	Beer	*65.9*	Parties	*69.2*	Enhancement	*40.43*
**Low-risk**							
**Drinkers**	*39.5*	Wine	*60.9*	Friend’s House	*53.7*	Social	*38.39*
*Men*	*45.9*						
*Women*	*37.8*	Cocktail	*58.2*	Home	*34.1*	Coping	*15.63*
**High-risk**							
**Drinkers**	*53.2*	Spirits	*31.0*	Disco	*32.1*	Conformity	*5.55*
*Men*	*49.2*						
*Women*	*54.6*	Others	*18.4*	Others	*11*		

**Table 5:** Percentage of correct answers to the knowledge about alcohol and FASD between groups of drinking-risk according to the AUDIT-C (World Health Organization, 2000. International guide for monitoring alcohol consumption and related harm. World Health Organization. https://apps.who.int/iris/handle/10665/66529) (accessed on 8 August 2021).

	**% of Correct Answers**		
	*No-Risk*	*Low-Risk*	*At-Risk*
***Item 1***	*79.5*	*88.8*	*87.6*
***Item 2***	*43.5*	*40.0*	*40.8*
***Item 3***	*44.8*	*46.4*	*46.0*
***Item 4***	*38.9*	*42.0*	*43.7*
***Item 5***	*48.3*	*61.2*	*69.9*
***Item 6***	*81.6*	*79.0*	*76.3*
***Item 7***	*12.4*	*8.2*	*6.8*
***Item 8***	*49.4*	*53.9*	*58.4*
***Item 9***	*44.0*	*47.0*	*49.2*
***Item 10***	*20.9*	*23.4*	*24.0*
***Item 11***	*44.4*	*46.8*	*50.8*
***Item 12***	*28.2*	*41.2*	*51.7*
***Item 13***	*37.2*	*46.1*	*45.0*
***Item 14***	*66.2*	*67.2*	*67.6*

## Data Availability

Data available on request due to restrictions eg privacy or ethical.
