# Correction: Messina et al. Knowledge and Practice towards Alcohol Consumption in a Sample of University Students. *Int. J. Environ. Res. Public Health* 2021, *18*, 9528

**DOI:** 10.3390/ijerph19148833

**Published:** 2022-07-20

**Authors:** Marisa Patrizia Messina, Gemma Battagliese, Alessio D’Angelo, Rosaria Ciccarelli, Fabiola Pisciotta, Luigi Tramonte, Marco Fiore, Giampiero Ferraguti, Mario Vitali, Mauro Ceccanti

**Affiliations:** 1 Department of Gynecology, Obstetrics and Urology, Sapienza University of Rome, 00185 Rome, Italy; marisapatrizia.messina@uniroma1.it (M.P.M.); alessio.dangelo96@gmail.com (A.D.); 2 Centro di Riferimento Alcologico della Regione Lazio, Mental Health Department, ASL Roma 1, 00185 Rome, Italy; gemma.battagliese@aslroma1.it; 3 Società Italiana per il Trattamento dell’Alcolismo e le sue Complicanze (SITAC), ASL Roma1, Sapienza University of Rome, 00185 Rome, Italy; r.ciccarelli@evodevo.it (R.C.); fabiola.pisciotta@uniroma1.it (F.P.); 4 Faculty of Medicine and Dentistry, Sapienza University of Rome, 00185 Rome, Italy; tramonte.1634054@studenti.uniroma1.it; 5 Institute of Biochemistry and Cell Biology (IBCN-CNR), 00185 Rome, Italy; marco.fiore@cnr.it; 6 Department of Experimental Medicine, Sapienza University of Rome, 00185 Rome, Italy; giampiero.ferraguti@uniroma1.it; 7 ASUR Marche, AV4, 60122 Ancona, Italy; vitalimario@yahoo.it

## Addition of an Author

**Gemma Battagliese** was not included as an author in the original publication [1]. The corrected Author Contributions Statement appears here:

**Author Contributions:** M.P.M., **G.B.**, A.D., R.C., L.T., M.F., F.P., M.F., G.F., M.V. and M.C.: Protocol/project development-Data analysis; M.P.M., **G.B.**, A.D., F.P., R.C., L.T.: Data collection or management; M.C., **G.B.**, G.F., A.D. and M.F.: Subtle Data Analysis; M.F., **G.B.**, M.C. and A.D.: Manuscript writing. All authors read and approved the final version of the manuscript.

## Correction of Affiliation

1. We have deleted the original affiliation 3 and added Gemma Battagliese’s affiliation “Centro di Riferimento Alcologico della Regione Lazio, Mental Health Department, ASL Roma 1, 00185 Rome, Italy”.

2. The right postcode of affiliation 7 is 60122.

The authors apologize for any inconvenience caused and state that the scientific conclusions are unaffected. The original publication has also been updated.
